# Food Insecurity and Not Dietary Diversity Is a Predictor of Nutrition Status in Children within Semiarid Agro-Ecological Zones in Eastern Kenya

**DOI:** 10.1155/2014/907153

**Published:** 2014-09-28

**Authors:** Zipporah N. Bukania, Moses Mwangi, Robert M. Karanja, Richard Mutisya, Yeri Kombe, Lydia U. Kaduka, Timothy Johns

**Affiliations:** ^1^Centre for Public Health Research, Kenya Medical Research Institute, P.O. Box 20752, Nairobi 00200, Kenya; ^2^Centre for Biotechnology Research and Development, Kenya Medical Research Institute, P.O. Box 54840, Nairobi 00200, Kenya; ^3^School of Dietetics and Human Nutrition, McGill University, Sainte Anne de Bellevue, QC, Canada H9X 3V9

## Abstract

Machakos and Makueni counties in Kenya are associated with historical land degradation, climate change, and food insecurity. Both counties lie in lower midland (LM) lower humidity to semiarid (LM4), and semiarid (LM5) agroecological zones (AEZ). We assessed food security, dietary diversity, and nutritional status of children and women. *Materials and Methods.* A total of 277 woman-child pairs aged 15–46 years and 6–36 months respectively, were recruited from farmer households. Food security and dietary diversity were assessed using standard tools. Weight and height, or length in children, were used for computation of nutritional status. *Findings.* No significant difference (*P* > 0.05) was observed in food security and dietary diversity score (DDS) between LM4 and LM5. Stunting, wasting, and underweight levels among children in LM4 and LM5 were comparable as were BMI scores among women. However, significant associations (*P* = 0.023) were found between severe food insecurity and nutritional status of children but not of their caregivers. Stunting was significantly higher in older children (>2 years) and among children whose caregivers were older. *Conclusion*. Differences in AEZ may not affect dietary diversity and nutritional status of farmer households. Consequently use of DDS may lead to underestimation of food insecurity in semiarid settings.

## 1. Introduction

Demonstration of the potential of food-based approaches that draw on local agriculture resources to improve food and nutrition security of small-holder farmers in sub-Saharan Africa is challenged by the lack of valid assessment tools able to recognize meaningful nutritional changes within short time frames [1–3]. Nutrition-sensitive and sustainable agriculture has found growing conceptual support [4, 5]; however, agricultural interventions within complex and dynamic ecological and socioeconomic environments typically fail to demonstrate significant improvement in direct measures of nutrient status [3].

Dietary diversity as a practical indicator of nutrient adequacy may be the most satisfactory proxy for nutritional quality both in comparing differences in available food resources and in response to dietary change [3, 5] but requires evaluation in relation to agricultural interventions. Individual dietary diversity score (IDDS) is useful as a proxy measure of the nutritional quality of an individual's diet [6] and as a reflection of nutrient adequacy [5, 7, 8]. Household dietary diversity score (HDDS) on the other hand is meant to reflect, in a snapshot form, the economic ability of a household access to variety of foods [5].

Nutrient intake, food choices, and dietary diversity are key determinants of nutritional status, especially in areas of high food insecurity such as the arid and semiarid lands (ASAL) of Eastern Kenya. Food and nutrition insecurity of public health importance affects the urban and rural poor in both developed and developing countries [9] while undernutrition contributes to increased mortality and morbidity in developing countries, with critical impacts on children's cognitive and physical development, quality of life, and lifetime productivity [10].

Diets recognized as monotonous, cereal-based, and lacking diversity that are characteristic of most developing countries, especially in Africa, are comprised of foods low in energy with few animal products, fruits, and vegetables [9, 11]. Consequently, inadequate quantities and unbalanced distribution of food types consumed by the household often result in nutritional deficiencies. Women of childbearing age and children under 5 years are particularly at risk of poor health; children have higher nutrient requirements for growth and are susceptible to infectious diseases such as diarrhoea and respiratory infections which can inhibit nutrient absorption and decrease appetite [12, 13].

## 2. Project Background

This paper presents baseline findings of dietary diversity and nutritional status of a study population participating in a 42-month (Canadian International Food Security Research Fund (CIFSRF) project). The project was implemented by Kenya Agricultural Research Institute (KARI) and McGill University, Canada. The two institutions partnered with Kenya Medical Research Institute, Ministry of Agriculture, Kenya, Cascade Development Organization, and FreshCo Seed Company to support the project in nutrition and health, agricultural extension, marketing dynamics, and seed production and supply. The general objective of the proposed project was to contribute to improved food security among women and men in hunger-prone communities. This was to be achieved by facilitating farmers to adopt proven agricultural technologies that enhance ecological resilience in the face of climate change.

The overarching theme of the study was to measure and evaluate any differences in the adoption, uptake, and performance of introduced agricultural technologies in the different agroecological zones (AEZs) and to understand factors that drive these differences. The agricultural productivity potential of land is one such factor that is inherently linked to nutritional and health status of rural communities that rely on subsistence farming. The two counties of Machakos and Makueni in Eastern Kenya, where this study was conducted, predominantly occupy semiarid lower midland (LM) agroecological zones (AEZs) 4 and 5 where farmers typically combine crop and livestock production under conditions of moderate intensity of land use. LM4 is a transitional zone between lower humidity and semiarid zone with a mean annual rainfall of 800 mm, whereas LM5 is semiarid with mean annual rainfall of 500 mm [14, 15]. In both zones, rain-fed dependent agriculture is the mainstay system, and thus directly affecting food production and subsistence-sourced dietary diversity.

We explored DDS as a predictor of nutritional status within the framework of baseline assessment and methods development for an agricultural technology-transfer project. While we predict correlations of DDS in this population characterized by high rates of childhood and adult malnutrition with measures of stunting, wasting, and underweight, as seen in published studies [16], we further tested the hypothesis that, in comparing agro-ecological zones that differ in agricultural potential and production, dietary diversity would reflect resultant differences in nutritional status. Specifically, we examined the impact of agro-ecological zones (AEZs) relative to known determinants on the dietary diversity of women and children in farming households.

## 3. Materials and Methods 

A descriptive study was carried out to assess the dietary diversity and nutritional status of women and children. Participants included children aged from 6 to 36 months and nonpregnant women of child-bearing age (15–46 years) from households of two counties of Machakos and Makueni in Eastern Kenya. At the outset, district agricultural officers were consulted in order to compile a list of registered farmer groups (FG) in the two counties. 72 farmers' groups from 119 villages met the inclusion criteria of being active in the last 6 months. Households were listed and data on age and sex of household members were collected to create a sampling frame of eligible households to recruit into the study. The proportion of FG membership whose households met the inclusion criteria of a woman-child pair where the woman was not pregnant but of child-bearing age (15–46 years) and the child of 6–36 months was determined. A simple random sampling technique was used to sample the households for participation in the study.

Lastly, 324 households were randomly selected from the sampling frame for inclusion in the survey with 307 successfully recruited and assessed. The nonresponses were due to absence. Thirty (30) households were excluded from the analysis due to missing or incomplete anthropometric measurements. Two hundred and seventy-seven, 134 child-woman pairs in Makueni and 143 in Machakos, were included in the study carried out in May and June 2012, a season described as “plenty” following harvest. Data collection and assessments were undertaken by a trained research team.

### 3.1. Sociodemographic Data

Demographic data included gender of the children, household size, and child's date of birth. Parents or guardians were asked to provide information on child's age which was confirmed using child immunization cards. Where cards were unavailable, the caretakers/guardians were asked to recall or use references to calendar events. Furthermore, information on household composition was collected.

### 3.2. Nutritional Status

Anthropometric measurements undertaken in all eligible respondents in the selected households included height/length and weight for children and women. Standard categories of nutritional status are reported according to WHO classification of anthropometric measurements cut-offs [17, 18]. Height and length were measured to the nearest 0.1 cm using the UNICEF wooden height and length boards while weight was assessed to the nearest 0.1 kg using a UNICEF Seca 762 classic mechanical medical weighing scale.

The nutritional status of the women of reproductive age (WRA) was assessed by body mass index (BMI) which was categorized according to World Health Organization standards [18].

### 3.3. Dietary Data

Household food consumption and dietary diversity data were collected with reference to both the child and the mother/caretaker/guardian. Respondents were requested to list all the foods consumed both at home and out of the home in the 24 hours preceding the interview. According to FAO, there are no established cut-offs points in terms of number of food groups to indicate adequate or inadequate dietary diversity for HDDS or IDDS [5]. Dietary diversity score (DDS) was calculated based on the number of different food groups consumed over a given reference period [19]. Mean scores or distribution of scores is, therefore, used for analysis [5].

Dietary diversity consisted of nine food groups for older children (24–36 months) and women. These foods included (1) starch staples, (2) grains and tubers, (3) dark green leafy vegetables, (4) other vitamin A rich fruits and vegetables, (5) other fruits and vegetables, (6) organ meats, (7) meat and fish, (8) eggs, and (9) legumes, nuts, and seeds. For the younger children 6–23 months, seven food groups are used [20]. Starchy staples, roots, and tubers were combined to form the grains, roots, and tubers food group, while meats, fish, and organ meats were combined and described as flesh foods group. Details about children's dietary intake were collected from their mothers or caregivers. Since the emphasis of this paper is on micronutrient intake as opposed to economic access, information on use of fats and oils has not been included in the scores.

### 3.4. Ethical Considerations

The study was approved by the Kenya Medical Research Institute's/National Ethical Review Committee. Prior to undertaking interviews, written consent was obtained from all adults, whereas for enrolment of children permission was obtained from their parents or guardians.

### 3.5. Statistical Analysis

Data entry was performed with Microsoft Access 2007. Data quality was maintained by quality checks during both data collection and entry (double entry) and further cleaning. All statistical analyses were carried out using IBM SPSS Statistics (version 20.0; IBM Corporation Software Group, NY, USA). Exploratory data analysis techniques were used to uncover the distribution structure of the study variables and identify outliers or unusually entered values. Distribution of continuous variables was tested for normality using Shapiro-Wilk test. All were found to follow normal distribution; therefore, independent *t*-test was used to test for mean differences between ecological zones. Chi-square test or Fisher exact test was used to test for independence in distribution of categorical variables (demographic characteristics, categorized nutritional variables, and dietary intake) between ecological zones. The same technique (Chi-square test or Fisher exact test) was used to assess the effect of various explanatory variables on nutritional status (stunting for children, underweight for WRA), with one explanatory variable assessed at a time. Odds ratios (OR) with corresponding 95% confidence intervals were estimated. In order to control for confounders, all explanatory variables that were associated with the nutritional status at *P* < 0.1 were considered together in a multiple logistic regression. Backward conditional method with removal at *P* < 0.05 was specified. Adjusted odds ratios (OR) with corresponding 95% confidence intervals were estimated.

## 4. Results

Table 1 presents characteristics of participating children and women in the survey. A total of 277 households were interviewed constituting 138 woman-child pairs from LM4 and 139 woman-child pairs from LM 5 AEZs. Mean age for WRA was 29.5 ± 0.4 (SE) years and 21.2 ± 0.5 (SE) months for the children (Table 1). Among the children, 49.8% were males and 50.2% were females.

The education levels and marital status of the WRAs were comparable in the two ecological zones. Less than 20% of the respondents reported having attained education above secondary levels but 81.2% had completed primary school. 87.3% of the WRA were married with household sizes averaging 7.0 members. This was also comparable in LM4 (mean household size 7.1) and LM5 (mean household size 6.8). Analyses of household food insecurity revealed that majority of the households (86.6%) were severely insecure. All variables were not significantly different between LM4 and LM5 (*P* > 0.05).

### 4.1. Nutritional Status and Dietary Diversity Score (DDS) in Women

The nutritional status of women between AEZs LM4 and LM5 showed no statistically significant difference (*P* > 0.05) (Table 2). The mean (SE) BMI for the study population was 21.9(0.2) Kg/m^2^ with thinness and overweight observed in 14.4% and 17.7% of the women, respectively. Interestingly, obesity levels exceeded those of wasting in both AEZs although these differences between zones were not significant (*P* = 0.05) (LM4: 18.8% versus LM5: 16.5%).

For the classification of dietary diversity, three categories were used in ranking DD score for women: low (1–3 food groups), medium (4-5 food groups), and high (≥6 food groups) [5, 6]. Women's dietary diversity score (DDS) was similar between LM4 and LM5 with high (≥6 food groups), medium (4-5 food groups), and low (1–3 food groups) percentages of 8.7%, 61.6%, and 29.7% compared to 5.8%, 63.3%, and 30.9%, respectively (Table 2).

### 4.2. Nutritional Status and Dietary Diversity (DDS) in Children

Children in AEZs LM4 and LM5 did not differ with statistical significance (*P* > 0.05) in mean *z*-scores (HAZ, WAZ, and WHZ) (Table 2). The mean *Z* scores (SE) for stunting, underweight, and wasting were −1.51(0.08), 0.78(0.07), and 0.02(0.07), respectively. Overall 33.8%, 11.6%, and 2.5% of the children aged 6–36 months were stunted, underweight, and wasted, respectively.

Stunting was slightly more prevalent in the semiarid LM5 (32.5%) compared to transitional LM4 (26.8%). However, proportions of underweight (LM4: 9.8% versus LM5: 10.4%) and wasting (LM4: 2.2% versus LM5: 2.9%) were nearly similar as shown in Table 2.

When disaggregated into age groups, significantly more older children (24–36 months) were stunted (39.7%) as compared to children younger than two years (29.6%; *P* < 0.05). Similarly, there was a greater burden of underweight and wasting in the older children (13.6% and 2.6%, resp.) than in the younger category (10.1% and 2.5%, resp.) although this was not statistically significant (*P* > 0.05).

Only 27.7% of children aged 6–23 months had adequate dietary diversity (a minimum of 4 food groups or more in the previous 24 hours). Within this same age group, more (76.6%) children in LM5 had inadequate dietary diversity compared to 68.3% in LM4. When DDS was calculated for the older children, upto 78.8% had low dietary diversity that was higher in LM4 (89.3%) than in LM5 (69.4%). A statistically significant difference in DDS between children aged 6–23 months and 24–36 months was observed (*P* = 0.008).

Using Pearson's correlation, a direct significant relationship was found between the DDS of the child and DDS of the caregiver/mother (*r* = 0.487; *P* = 0.001); as the DDS of the mother increases, the DDS of the child increases.

### 4.3. Nutritional Status of Children and Women in relation to Selected Sociodemographic Characteristics

Table 3 presents the nutritional status in children in relation to selected sociodemographic characteristics. Age 31–46 years among women was found to be significantly associated with stunting in children. A higher proportion of the older children (24–36 month) were stunted (39.7%) compared to their younger counterparts (29.6%) (OR = 1.57; 95% CI: (0.95–2.59); *P* = 0.081). For children whose caregivers were older (31–46 years) a significantly higher proportion was stunted compared to children whose caregivers were relatively young (15–30 years) (OR = 1.81; 95% CI: (1.08–3.02); *P* = 0.024). No significant associations were found with age of child, sex of child, education level of the woman/caregiver, marital status of the woman/caregiver, ecological zones, DDS of child, household size, and nutritional status of the woman. Upon controlling for confounders, binary logistic regression revealed older age in woman/caregiver (AOR = 1.77; 95% CI: (1.05–2.97); *P* = 0.032) and severe food insecurity (AOR = 2.90; 95% CI: (1.16–7.25); *P* = 0.023) to be strongly associated with stunting in children.

Table 4 presents the nutritional status of women respondents in relation to selected sociodemographic. Contrary to expectation, no significant association was found between nutritional status of the mother and dietary diversity or food security. Low education level among women could have an implication on their own nutritional status since our findings show a marginal significant association (OR = 3.23; 95% CI: (0.96–10.9) (*P* = 0.05)).

## 5. Discussion

Agricultural potential as defined by the ecological zones did not affect the dietary diversity (DDSs) and nutritional status of households. Household characteristics that posed as confounders did not differ significantly between LM4 and LM5 (Table 1). However, the overall mean household size of 7.0 was found to be much higher than the national mean of 4.2 as reported by the Kenya Demographic Health Survey [16]. A household was defined as members of a community that live as a cohesive unit and eat from the same “pot.” Machakos and Makueni counties are historically food insecure and characterized by land degradation, cycles of drought and famine, and reliance on food aid [20, 21]. The large household size reported may, therefore, represent a coping strategy in the face of persistent food insecurity through leveraging food access by increasing the ratio of number of persons per pot [22].

### 5.1. Nutritional Status

Individual dietary diversity has been described [6] as a proxy measure of nutrition/dietary quality and can be a good indicator of overall household food security and positively associated with nutritional status in children [23]; this would, therefore, explain why long term poor dietary diversity is likely to be reflected in stunting. We observed an association between severe food insecurity and stunting (AOR = 2.90; 95% CI: (1.16–7.25); *P* = 0.023) but not with wasting and underweight. Stunting or chronic malnutrition is usually an indication of long term deprivation of nutrients in children. Stunting remains a problem of greater magnitude than underweight or wasting, and it more accurately reflects nutritional deficiencies and illness that occur during the most critical periods for growth and development in early life [24].

The consequences of seasonal changes on the nutritional status of adults have been well documented although dietary diversity scores are usually measured during a single period of the year and their seasonal variations remain largely unknown [23]. Our findings showed no association between DDS and nutritional status of the women in this study; however, almost two thirds of the women (62.3%) had medium and less than 8% had high DDS at the time of this survey. This could be attributed to seasonality since our survey was undertaken in May/June at a season dubbed “season of plenty” immediately after harvest. It would be useful to reconsider measuring dietary diversity during the food shortage season, to understand the seasonal differences.

The lower the education level, the higher the prevalence of underweight among women. These findings are similar in many populations where the level of education is directly related with obesity especially in women [25]. Education, therefore, appears as a protective factor for the underweight women but as a risk factor for the overweight and obese women [26]. More immediately, at least, increasing the options of a household as mediated by factors, such as women's education, to source for other preferred food choices may have greater impact on nutritional status. FAO analysed data from 48 low income countries and found that primary education is a key determinant of food security in low income communities. FAO further emphasizes that female education has a direct additional benefit to nutritional status [27].

### 5.2. Dietary Diversity

In addition to improved outcomes in child birth weights, anthropometric status and improved haemoglobin status, dietary diversity is highly correlated with caloric and protein adequacy [6]. Our findings indicate moderate DDS among the women however, not statistically significant (*P* = 0.639). Most of the children on the other hand had low diversity 72.3% and 78.8% 6–23 and 24–36 months old respectively, mostly consuming less than 4 food groups in a day. Statistically significant differences in DD in children were identified (*P* = 0.008) between the ecological zones. However, it has been found that although there is a definite and strong association between mother and child DD, mother child agreement in intake of foods from different food groups may not necessary allow using mothers DD for calculating the child's DD [3].

### 5.3. Strengths and Limitations

The use of experienced research assistants ensured accuracy in data collection. Moreover, the customization of the data collection tool to suit the study population ensured that all the relevant foods were listed to guide research assistants in probing for all foods possibly consumed in the household including alternative sources foraged from the wild with the potential of augmenting or negating differences in DD between the AEZs. Careful field supervision also allowed us to have accurate data collection with minimal missing variables.

One limitation of our study was that dietary data was collected for only one recall. This can potentially limit the true dietary diversity on different days of the week. Self-reporting of dietary data in a food insecure population where respondents might assume that underreporting of consumption offered a possible opportunity for food support could introduce a potential bias. An improvement on the dietary diversity data tool could help collect information on food varieties specific to food groups. This is an aspect that may need consideration in future surveys. Additionally, the survey was undertaken in one season and at a time of plenty. More DDS done in different seasons may better explain the true dietary diversity of the study population.

## 6. Conclusion 

Child nutritional status was dependent on household food security and not dietary diversity. Moreover, differences in AEZ did not affect dietary diversity and nutritional status of farmer households. This may be attributed to seasonal variation that has been shown to significantly impact the estimation of food security status. DDS may therefore not be the preferred predictor for nutritional status, and further interrogation of its applicability in diverse settings is necessary. Conversely, the caregivers' education levels strongly correlated with the nutritional status of the children. In addressing food insecurity, enhancing women's nutrition knowledge in combination with nutrition-sensitive agricultural interventions, can be expected to significantly improve food security and nutritional status of children and women.

Certainly nutrition-sensitive agriculture that focuses on increasing locally-sourced diversity is more likely to achieve impact in conjunction with population wide nutrition education directed especially to caregivers that emphasizes the need for diversified diets. Increased household income is a major contributor to improved nutrition, although on its own it is not enough; it may be inefficient or ineffective if women have no level of control. Women are more likely to spend the income they control on health, food, and education of their children [28]. Additionally, improvements in mechanization focused on agricultural production in the study area can free women of heavy workloads, thus creating more time to care for their children and families.

## Figures and Tables

**Table 1:** Characteristics of women and children in farmer households in lower midland ASAL regions of Eastern Kenya.

Characteristic WRA	Total (*n* = 277)	LM4 (*n* = 138)	LM5 (*n* = 139)	*P* value
Mean (SE) years	29.5 (0.4)	29.3 (0.5)	29.6 (0.6)	0.675
15–29 years %	56.7	58.7	54.7	0.463
30–39 years %	33.9	34.1	33.8	
40–46 years %	9.4	7.2	11.5	
Marital status				
Never married %	9.6	9.0	10.2	0.795
Married %	86.0	87.3	84.7	
Separated/widowed/divorced %	4.4	3.7	5.1	
Education level				
Primary level and below %	81.2	81.9	80.4	0.878
Secondary level and below %	18.8	18.1	19.6	
Characteristics, children 6–36 months				
Gender (%)				
Girls %	50.2	53.6	46.8	0.254
Boys %	49.8	46.4	53.2	
6–23 months %	57.4	59.4	55.4	0.498
24–36 months %	42.6	40.6	44.6	
Age in months				
Mean (SE) months	21.2 (0.5)	20.8 (0.7)	21.5 (0.7)	0.446
Mean (SE) 6–23 months	15.0 (0.4)	15.0 (0.5)	15.0 (0.5)	0.986
Mean (SE) 24–36 months	29.4 (0.3)	29.2 (0.4)	29.6 (0.4)	0.525
Household characteristics				
Family size				
Mean household size (SE)	7.0 (0.1)	7.1 (0.2)	6.8 (0.2)	0.274
3-4 people %	11.4	9.6	13.1	0.410
5-6 people %	37	40.4	33.6	
≥7 people %	51.6	50.0	53.3	
Household food security				
Food secure %	6.5	5.1	8.0	0.173
Moderately insecure %	6.9	9.4	4.3	
Food insecure %	86.6	85.5	87.7	

SE: standard error; LM: lower midlands agroecological zones.

**Table 2:** Nutritional status and dietary diversity score in women and children by agroecological zones.

	Total (*n* = 277)	LM4 (*n* = 138)	LM5 (*n* = 139)	*P* value
*Nutritional status of women *				
Mean (SE) weight (Kg)	54.3 (0.6)	54.2 (0.9)	54.4 (0.8)	0.863
Mean (SE) height (cm)	157.4 (0.4)	157.4 (0.5)	157.3 (0.6)	0.912
Mean (SE) BMI (Kg/m^2^)	21.9 (0.2)	21.8 (0.3)	22.0 (0.3)	0.738
Wasted %	14.4	16.7	12.2	0.447
Overweight %	17.7	18.8	16.5	
Normal %	67.9	64.5	71.2	
*Dietary diversity in women *				
Dietary diversity score (DDS)				
Low DDS %	30.3	29.7	30.9	0.639
Medium DDS %	62.5	61.6	63.3	
High DDS %	7.2	8.7	5.8	
*Nutritional status in children *				
Mean *Z* scores in children				
Mean (SE) HAZ *Z*-score	−1.51 (0.08)	−1.44 (0.12)	−1.59 (0.12)	0.392
Mean (SE)WAZ *Z*-score	−0.78 (0.07)	−0.76 (0.09)	−0.80 (0.10)	0.783
Mean (SE) WHZ *Z*-score	0.02 (0.07)	−0.02 (0.09)	0.05 (0.10)	0.596
Stunting %	33.8	31.6	36.0	0.445
Underweight %	11.6	10.9	12.2	0.723
Wasting %	2.5	2.2	2.9	1.000
6–23 months				
Stunting %	29.6	26.8	32.5	0.436
Underweight %	10.1	9.8	10.4	0.894
Wasting %	2.5	2.4	2.6	1.000
24–36 months				
Stunting %	39.7	38.9	40.3	0.875
Underweight %	13.6	12.5	14.5	0.749
Wasting %	2.6	1.9	3.2	1.000
*Dietary diversity score in children *				
6–23 months				
Inadequate %	72.3	68.3**	76.6	0.241
Adequate %	27.7	31.7	23.4	
24–36 months				
Low %	78.8	89.3**	69.4	0.008
Medium %	21.2	10.7	30.6	

*Difference in DDS between 6–23 and 24–36 months is significant (*P* < 0.05).

**Table 3:** Nutritional status of children in relation to selected sociodemographic and economic characteristics.

Characteristics	Stunting status				Bivariate analysis				Multivariate analysis			
	Stunted		Normal		OR	95% CI		*P* value	AOR	95% CI		*P* value
	*n*	%	*n*	%		Lower	Upper			Lower	Upper	
Age of the child in months												
6–23	47	29.6	112	70.4	1.00							
24–36	46	39.7	70	60.3	1.57	0.95	2.59	0.081				
Sex of the child												
Male	49	36.0	87	64.0	1.22	0.74	2.01	0.443				
Female	44	31.7	95	68.3	1.00							
Age in years of the woman												
15–30	51	29.0	125	71.0	1.00				1.00			
3–46	42	42.4	57	57.6	1.81	1.08	3.02	**0.024**	1.77	1.05	2.97	**0.032**
Education level of the woman												
Primary and below	78	35.1	144	64.9	1.47	0.75	2.88	0.259				
Secondary and above	14	26.9	38	73.1	1.00							
Marital status of the woman												
Never married	9	34.6	17	65.4	0.74	0.18	3.02	0.676				
Married	76	32.9	155	67.1	0.69	0.21	2.23	0.532				
Separated/divorced/widowed	5	41.7	7	58.3	1.00							
Ecological zone												
LM4	43	31.6	93	68.4	1.00							
LM5	50	36.0	89	64.0	1.22	0.74	2.00	0.445				
Household with severe food insecurity												
Yes	87	36.6%	151	63.4%	2.98	1.19	7.42	**0.015**	2.90	1.16	7.25	**0.023**
No	6	16.2%	31	83.8%	1.00				1.00			
DDS of children												
Inadequate	75	36.4	131	63.6	1.62	0.88	2.98	0.117				
Adequate	18	26.1	51	73.9								
Household size												
3-4 people	13	39.4	20	60.6	1.16	0.53	2.52	0.709				
5-6 people	29	29.0	71	71.0	0.73	0.42	1.27	0.261				
≥7 people	51	35.9	91	64.1	1.00							
*NS of the woman												
Underweight	12	30.0	28	70.0	0.88	0.36	2.18	0.789				
Normal	65	34.9	121	65.1	1.11	0.57	2.16	0.764				
Overweight	16	32.7	33	67.3	1.00							

*Age of woman in years, nutritional status significant at *P* < 0.05.

**Table 4:** Nutritional status of women respondent in relation to selected sociodemographic and economic characteristics.

Characteristics	Underweight		Normal/overweight		OR	95% CI		*P* value
	*n*	%	*n*	%		Lower	Upper	
Age in years								
15–30	28	15.8	149	84.2	1.38	0.67	2.85	0.385
31–46	12	12.0	88	88.0	1.00			
Education level*								
Primary and below	37	16.5	187	83.5	3.23	0.96	10.9	0.050
Secondary and above	3	5.77	49	94.2	1.00			
Marital status								
Never married	3	11.5	23	88.5	1.43	0.13	15.4	0.766
Married	36	15.5	197	84.5	2.01	0.25	16.1	0.510
Separated/divorced/widowed	1	8.33	11	91.7	1.00			
Ecological zone								
LM4	23	16.7	115	83.3	1.44	0.73	2.82	0.294
LM5	17	12.2	122	87.8	1.00			
Dietary diversity score								
Low	12	14.3	72	85.7	0.94	0.24	3.72	0.935
Medium	25	14.5	148	85.5	0.96	0.26	3.51	0.947
High	3	15.0	17	85.0	1.00			
Household food security								
Food secure	1	5.56	17	94.4	1.00			
Moderately food insecure	3	15.8	16	84.2	3.19	0.30	33.89	0.336
Food insecure	36	15.1	203	84.9	3.01	0.39	23.36	0.291
Household size								
3-4 people	4	11.8	30	88.2	1.00			
5-6 people	13	13.0	87	87.0	1.12	0.34	3.70	0.852
≥7 people	23	16.1	120	83.9	1.44	0.46	4.47	0.531

*Education level of woman.
